# Chiroptically Active Multi-Modal Calcium Carbonate-Based Nanocomposites

**DOI:** 10.3390/nano14010100

**Published:** 2023-12-31

**Authors:** Fearghal C. Donnelly, Finn Purcell-Milton, Eoin Caffrey, Lorenzo Branzi, Shelley Stafford, Faisal Ali Alhammad, Olan Cleary, Munirah Ghariani, Vera Kuznetsova, Yurii K. Gun’ko

**Affiliations:** 1 School of Chemistry, Trinity College Dublin, D02 PN40 Dublin, Irelandbranzil@tcd.ie (L.B.); alhammaf@tcd.ie (F.A.A.);; 2 BiOrbic Bioeconomy SFI Research Centre, University College Dublin, D04 F438 Dublin, Ireland; 3 Chemical & BioPharmaceutical Science, Technological University Dublin, Grangegorman, D07 H6K8 Dublin, Ireland; 4 School of Physics, Trinity College Dublin, D02 PN40 Dublin, Ireland

**Keywords:** quantum dots, core–shell nanostructures, chiroptical activity, photoluminescence, calcium carbonate

## Abstract

The development of multimodal nano- and micro-structures has become an increasingly popular area of research in recent years. In particular, the combination of two or more desirable properties within a single structure opens multiple opportunities from biomedicine, sensing, and catalysis, to a variety of optical applications. Here, for the first time, we report the synthesis and characterization of multimodal chiroptically active CaCO_3_ nanocomposites. These composites have been prepared by a modified microemulsion method in the presence of an amino acid (cysteine). Following this, additional modalities have been introduced by loading the composites with luminescent nanoparticles or doping with Eu^3+^ ions. The luminescent composites have been produced by the incorporation of CuInZnS/ZnS or CdSe@ZnS/ZnS core/shell quantum dots, or via doping with trivalent europium. In this manner, we have produced chiroptically active composites with orange, green, and red luminescence. Overall, this work demonstrates the unique advantage and potential of our approach and new class of chiroptically active CaCO_3_ nanocomposites, which display tunable functionality to specific requirements via the incorporation of desired ions, nanoparticles, and chirality of the structure.

## 1. Introduction

Calcium carbonate is amongst the oldest and most widely studied materials in existence. Naturally occurring as limestone, marble and chalk, it provides a major source of global CO_2_ sequestration [1] as well as being an important biomineral responsible for a variety of animal and marine life functions. Industrially, it finds use in the construction industry, in the production of high gloss paper [2], and as white pigment in paint [3], as well as in various fields of biomedicine in both drug delivery and in calcium supplementation. It exists in three anhydrous forms, which are, in order of decreasing stability: calcite, aragonite, and vaterite, and possess three further hydrated forms: amorphous calcium carbonate (ACC), ikaite, and monohydrocalcite [4]. Additionally, we believe its biocompatibility and robust nature make it an ideal substrate to produce novel multi-modal materials.

A particularly interesting research area is the development of multimodal nano- and micro-composite structures [5,6,7] that can be specifically tailored to various applications including in sensing, photonics, catalysis, and drug delivery [7,8,9,10,11]. These structures have been demonstrated in a variety of materials including silica, various polymeric materials, and CaCO_3_ offering the specific advantages of bioavailability in tandem with favorable optical properties. In the production of CaCO_3_ composites, a variety of microemulsions are frequently used, which generally rely on micelles to encapsulate the required moiety within the CaCO_3_ matrix. These methods are highly effective and have found success, for example, in drug delivery. Oil-in-water (O/W) microemulsions, for example, have recently been used to encapsulate the anticancer drug doxorubicin (DOX) through a high-pressure homogenization (HPH) system and showed high selectivity with minimal associated toxicity in healthy tissue [12]. However, the CaCO_3_ composites’ applications are not limited to drug delivery: they have been recently been used as vaccine adjuvants [13], while Liu et al. illustrated their potential for catalysis [14]. Nanocomposites based on Eu^3+^-doped CaCO_3_ have also been prepared using a traditional carbonation method and then calcined to obtain Eu^3+^-doped CaO phosphors [15].

Chiral compounds, i.e., those that exist in two non-superimposable mirror-image forms known as enantiomers, are of exceptional importance to a variety of fields including chemistry, biology, and medicine. The enantioselectivity of chiral substances is highly useful in a variety of applications from enantiomeric separation to asymmetric catalysis as well as in biochemistry and other applications. In addition, many biomineralization processes are often heavily reliant on chirality, including the formation of various CaCO_3_-based structures.

Luminescent nanomaterials also possess an ever-increasing research interest. These materials find uses in several fields, from LEDs and photovoltaics to bioimaging and sensing. Two classes of luminescent nanomaterials, semiconductor quantum dots (QDs) and lanthanide-doped nanomaterials, present a particular interest. QDs are an important type of luminescent nanomaterials with a variety of controlled morphologies and associated luminescence properties [16]. Core–shell QDs, which are used in this work, involve the coating of a semiconductor with a shell of a second bandgap semiconductor, which allows the further tuning of their optical properties [17]. Lanthanide (e.g., Eu and Tb)-doped nanostructures are also a highly effective luminescent species and have been investigated extensively. It was demonstrated that the lanthanide doping of inorganic materials results in highly stable materials with large Stokes shifts, narrow emission peaks, and some of the longest photoluminescent lifetimes available [15,18,19,20,21,22,23,24]. QD-loaded inorganic structures have been used for numerous applications including but not limited to biosensing [25], as well as optical microresonators [26] and white light emitters [27]. Lanthanide-doped composites, meanwhile, have found applications in biological studies [28], upconversion [29], catalysis [30], and optoelectronics [21]. While Eu^3+^ doping of CaCO_3_ has been studied and will be discussed below, the only few examples that exist of quantum dot loading into CaCO_3_ composites have shown a preservation of their individual properties within the CaCO_3_ matrix.

The main aim of this work is to develop a new class of chiroptically active multimodal CaCO_3_ nanocomposites loaded with luminescent nanoparticles. These CaCO_3_-based composites have been prepared via a modified chiral ligand-assisted microemulsion approach. In addition, the composites have been loaded with a large variety of luminescent active components of remarkable importance in the literature. For this purpose, we focused our investigation on well-known luminophores such as cadmium selenide QDs shelled with ZnS (CdSe@ZnS/ZnS QDs) [31] and quaternary copper indium zinc sulfide passivated with ZnS shells (CuInZnS/ZnS QDs) [32,33,34,35] as well as Eu^3+^ ions resulting in new multimodal luminescent microstructures with a range of potential applications.

## 2. Experimental Section

### 2.1. Starting Materials

Calcium chloride dihydrate (CaCl_2_·2H_2_O, ≥99.0%), sodium carbonate (Na_2_CO_3_, ≥99.5%, ACS reagent, Barcelona, Spain), Eu(NO_3_)_3_·6H_2_O (99.9%), cadmium acetate (≥99.99%) polyvinylpyrrolidone (PVP) (MW 360,000), sodium dodecyl sulfate (SDS), copper iodide (CuI, 99.99%), indium acetate (In(OAc)_3_, 99.99%), 1-dodecanethiol (1-DDT, 98%), zinc stearate (technical grade), zinc acetate dehydrate (99.999%), 1-octadecene (ODE, ≥95%), trioctylphosphine (TOP, 97%), diphenylphosphine (DPP, 98%), oleic acid (OA, ≥99%), cetyltrimethylammonium bromide (CTAB, 99%), L-cysteine (98%), L- (≥99%), and D-proline (99%) were supplied by Sigma Aldrich, St. Louis, MO, USA (Merk, Darmstadt, Germany) and used without further purification. All solvents used were supplied by the Trinity College Dublin Hazardous Materials facility.

### 2.2. Synthesis of Chiroptically Active CaCO_3_ Composites

SDS (0.5 g; 90 mmol), PVP (0.5 g; 34.7 mmol), and L-cysteine (0.6 g; 50 mmol) were added to 50 mL of Na_2_CO_3_ (0.1 M) and allowed to stir for 1 h. SDS (0.721 g; 50 mmol) was added to a 50 mL solution of CaCl_2_ and stirred for 15 min before being added to the Na_2_CO_3_ solution. The resulting suspension was stirred for a further 1 h. The product was subsequently centrifuged and washed with Millipore water and ethanol before drying at 80 °C for 24 h. This process has resulted in yields in excess of 70%.

### 2.3. Synthesis of Colloidal CuInZnS/ZnS QDs

The quantum dots CuInZnS/ZnS were prepared according to previously published and modified by us procedures [35].

#### 2.3.1. Synthesis of Colloidal CuInS QDs

A total of 0.2 mmol (0.0.358 g) of CuI, 0.6 mmol (0.1168 g) of In(OAc)_3_, and 20 mL of 1-DDT were mixed in a 100 mL 3-neck flask. The flask was degassed for 30 min at 80 °C, and then the atmosphere was switched to argon. Under argon flux, the reaction temperature was increased to 215 °C, and held at this temperature for 10 min. Then, the heating mantle was removed and the reaction vessel was cooled to room temperature naturally. Without purification, the crude solution was ready for the synthesis of CuInZnS alloyed QDs.

#### 2.3.2. Synthesis of Colloidal CuInZnS QDs

The Zn precursor solution was prepared by dissolving 0.6 mmol (0.3768 g) of zinc stearate in 8 mL of ODE. A total of 2 mL of TOP was injected into the reaction flask under argon gas. The mixture was heated to 100 °C until a clear precursor solution was obtained. Then, CuInZnS QDs were prepared through in situ cation exchange of the preformed CuInS QDs. The crude CuInS QD solution was degassed at 115 °C for 30 min to remove volatiles that were produced during the reaction. Then, the atmosphere was switched to argon and the temperature was set to 120 °C. When the temperature reached the target temperature, the zinc precursor solution was added drop-wise with a syringe that was set on a syringe pump over 30 min (injection rate = 20 mL/h) to the reaction vessel. When the injection was finished, the temperature was increased to 200 °C and maintained at that temperature for 90 min before cooling to RT. Without cleaning, the sample was ready for ZnS shell coating.

#### 2.3.3. Synthesis of CuInZnS/ZnS QDs

A total of 4 mmol (2.53 g) of zinc stearate was dissolved in 10 mL of oleic acid, 10 mL of 1-DDT, and 20 mL of ODE. The mixture was degassed for 2 h at 30 °C under stirring and then the reaction vessel was flashed with argon. The crude CuInZnS QDs solution in the reaction vessel was degassed for 30 min at 120 °C to remove volatiles that produced during the reaction. Under an argon atmosphere, the temperature was increased to 220 °C. When the temperature reached 200 °C, the above precursor solution was injected into the reaction vessel with a syringe pump (the injection rate was ~4 mL/h for the volume of 40 mL), with the injection taking 10 h. After the injection, the solution was stirred for another 1 h at 220 °C. Then, the heating mantle was removed and the reaction vessel was cooled to RT naturally. Hot ethanol, toluene, and chloroform were used to purify the sample. The obtained clean CuInZnS/ZnS QDs were stored in chloroform in the fridge.

### 2.4. Synthesis of Colloidal CdSe@ZnS/ZnS QDs

CdSe@ZnS/ZnS QDs were prepared according to previously published and modified by us procedures [31].

A total of 0.7 mmol of Cd acetate and 1.7 mmol of Zn oxide were mixed with 3.5 mL of OA. This mixture was heated to 150 °C under an argon atmosphere. Then, 10 mL of 1-octadecene (ODE) was added into the reactor at that temperature, and the mixture was heated to 310 °C. An anionic stock solution was prepared by dissolving 2.5 mmol of Se and 2.5 mmol of S in 5 mL of TOP. Then, 2.0 (Se + S) mL of TOP was quickly injected into the above hot mixture and the reaction proceeded at that temperature for 10 min for the growth of CdSe@ZnS QDs. For producing CdSe@ZnS/ZnS QDs through an additional ZnS coating, 0.8 mmol of S dissolved in 2 mL of ODE was consecutively introduced into the above growth solution of CdSe@ZnS QDs at 310 °C, followed by a holding time of 12 min. Next, a Zn precursor stock solution, prepared by 1.43 mmol of zinc acetate dehydrate dissolved in a mixture of 1 mL of oleic acid and 4 mL of ODE, was injected at 310 °C, after which the temperature of the reaction became lowered to 270 °C. The sulfur stock solution was prepared from 4.8 mmol of S dissolved in 5 mL of TOP. Then, the sulfur precursor was introduced drop-wise and the additional ZnS shelling proceeded at 310 °C for 20 min. The resulting CdSe@ZnS and CdSe@ZnS/ZnS QDs were precipitated by adding excess ethanol and repeatedly washed with a solvent combination of hexane/ethanol (1:4 in volume ratio) by centrifugation, and the purified QDs were redispersed in chloroform.

### 2.5. Synthesis of Quantum Dot-Loaded CaCO_3_ Composites

To produce quantum dot-loaded composites, the CdSe@ZnS/ZnS or CuInZnS/ZnS QDs (0.1 mL aliquot in chloroform) were added to 50 mL of Na_2_CO_3_ (0.1 M) solution along with SDS (0.5 g; 90 mmol), PVP (0.5 g; 34.7 mmol), and L-cysteine (0.6 g; 50 mmol). The mixture was stirred for 1 h. Then, SDS (0.721 g; 50 mmol) was added to a 50 mL solution of CaCl_2_ and stirred for 15 min before being added to the Na_2_CO_3_ and QD solution above. The resulting suspension was stirred for a further 1 h. The precipitated product was subsequently centrifuged and washed with Millipore water and ethanol before drying at 80 °C for 24 h. This process resulted in yields of the product in excess of 70%.

### 2.6. Synthesis of Europium-Loaded CaCO_3_ Composites

Europium calcium carbonate composites were prepared similarly to the QD-loaded composites above using Eu(NO_3_)_3_·6H_2_O (0.223 g; 5 mmol) in 10 mL of Millipore water instead of QDs. The product was isolated by centrifugation, washed with Millipore water and ethanol and then dried at 80 °C for 24 h.

### 2.7. Instrumentation and Equipment

All diffuse reflectance (DR) UV-Vis spectroscopy measurements were acquired using a Perkin Elmer Lambda 1050 UV-Vis-NIR spectrophotometer (Perkin Elmer, Waltham, MA, USA) operating in transmission mode, with a 150 mm InGaAs integrating sphere. Samples were prepared for DR UV-Vis by drop-casting some of the colloidal suspension onto a VWR super premium microscope slide cut to size, followed by solvent evaporation using a hot plate at 100 °C. The spectra were recorded within the wavelength range of 200–400 nm.

Solid state diffuse reflectance circular dichroism (DRCD) absorption spectra were measured using a Jasco J-815 CD spectrometer (Jasco, Dublin, Ireland) with a DRCD-575 integrating sphere and BaSO_4_ internal background in the wavelength range from 200 to 400 nm.

X-ray diffraction (XRD) patterns were recorded on a Bruker D2 Phaser 2nd Gen benchtop diffractometer (Bruker, Billerica, MA, USA) using Cu Kα radiation (λ = 1.5418 Å) across a 2θ range of 15 to 55° with a step–size of 0.01° at 1 s/step using a zero–background Si sample holder. Unit cell parameters and crystallite size were determined by Rietveld refinement of the obtained patterns performed using FullProf v5.10 implemented in EXPGUI. The starting point for refinements was (*R* − 3*c*; *a* = 4.99; *c* = 17.0615).

Scanning electron microscopy (SEM) was carried out on a Zeiss Ultra Plus Scanning Electron Microscope (Zeiss, Jena, Germany) operating at 3.50 kV.

Transmission electron microscopy (TEM) was carried out on an FEI Titan Transmission Electron Microscope (FEI, Hillsboro, OR, USA) operating at 300 kV.

Focused ion beam (FIB) microscopy was carried out using a Zeiss AURIGA Focused Ion Beam Scanning Electron Microscope.

Mercury porosimetry was carried out using an Autoscan-33 Porosimeter (Quantachrome, Hook, UK).

FT-IR transmission spectra were recorded on a Bruker Tensor II FT-IR spectrometer using a diamond UATR. The spectra were recorded in the range from 400 to 4000 cm^−1^.

Photoluminescence (PL) spectra were recorded using a Horiba FluorMax-4 (Horiba, Kyoto, Japan) in phosphorimetry mode. Steady-state measurements were made using a 0.1 ms delay, a sample window of 25 ms, a flash count of 200, and an increment of 1 nm, using a solid-state powder. Excitation spectra were measured using excitation and emission slits of 2 nm and 2 nm while emission spectra were measured using excitation and emission slits of 3 nm and 3 nm. The powder samples were mounted on a solid sample holder, and instead, CdSe@ZnS/ZnS QDs and CuInZnS/ZnS QDs were characterized as colloidal solution in toluene.

Photoluminescence lifetime and time resolved emission spectra measurements were performed using a time-correlated single-photon counting (TCSPC) spectrometer (Fluorolog 3 Horiba Jobin Yvon) and a range of different semiconductor diode lasers (295 nm, 340 nm, and 393 nm “Nano LED”—HORIBA Jobin Yvon) with a pulse duration shorter than 1 ns for excitation. Eu^3+^ phosphorescence lifetime decay was determined using the decay by window mode; in addition, it was carried out using a flash delay of 0.05 ms, a flash count of 200, and excitation/emission slits of 3 nm and 3 nm.

EDS analysis was performed using an 80 mm^2^ window Oxford Instrument (Abingdon-on-Thames, UK) Xmax EDX detector and working with an EHT of 15 KeV.

## 3. Results and Discussion

### 3.1. Synthesis of Multi-Modal CaCO_3_ Nanocomposites

**Figure 1:**
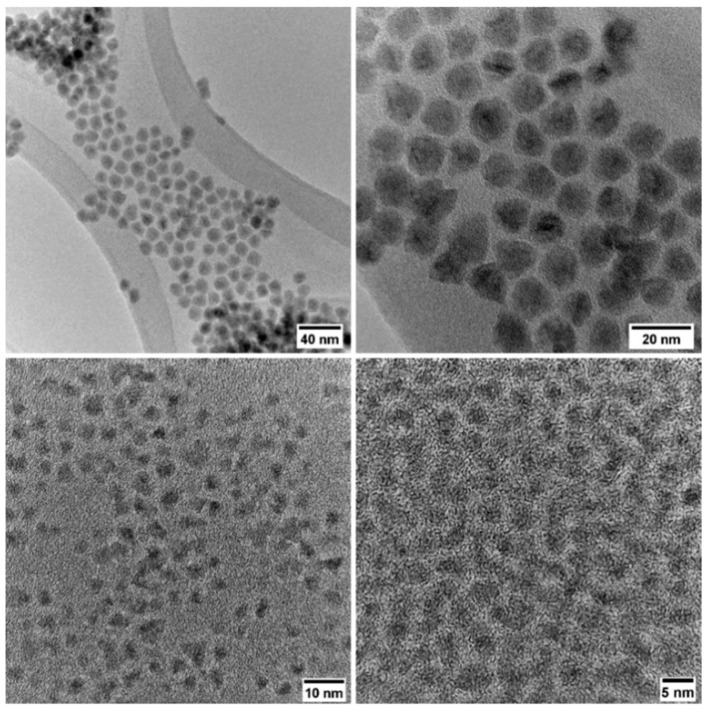
TEM images of CdSe@ZnS/ZnS QDs (**top**) and CuInZnS/ZnS QDs (**bottom**) that were used for the preparation of the calcium carbonate composites.

Two different types of nanoparticles have been used for incorporation into CaCO_3_ composites, namely CuInZnS/ZnS QDs and CdSe@ZnS/ZnS QDs. Both possess core–shell structures with a ZnS shell and with CuInZnS and CdSe@ZnS cores, respectively. CdSe@ZnS means that CdSe is partially alloyed with ZnS, resulting in an improvement of QD optical properties. Both types of QDs have been synthesized using previously reported methods described in the Section 2 below. Cadmium selenide QDs are characterized by their narrow emission peak, which allows them a high color purity, which is ideal for application in QLED. Green-emitting cadmium selenide QDs have been produced according to the previous report from Lee and co-workers [31]. The authors observed superior optical properties as well as high stability for the CdSe QDs stabilized by a ZnS chemical composition gradient shell and a further coated with a second ZnS shell layer producing a giant 12.7 nm CdSe@ZnS/ZnS QDs. The role of large-band gap semiconductors such as ZnS is critical in obtaining a type-I band alignment and guaranteeing the consequent confinement of both the photo-generated electron and hole in the CdSe core, while minimizing the lattice mismatch. On the other hand, cadmium-free quaternary QDs such as copper indium zinc sulfide are of great interest due to their superior biocompatibility for the design of luminescent solar concentrators and white LED production [32,33,34]. Copper indium zinc sulfide QDs passivated with a zinc sulfide shell (CuInZnS/ZnS QDs) are produced using a protocol from Zhang and co-workers [35]. This approach is based on the isolation of a ternary copper indium sulfide core, the production of an alloyed quaternary core via ion exchange reaction in the presence of Zn^2+^, and finally ZnS passivation to produce a core–shell QDs. These specific CuInZnS/ZnS and CdSe@ZnS/ZnS QDs were chosen for their monodispersity and favorable optical properties as well as their broad range of applications. The TEM images of CdSe@ZnS/ZnS and CuInZnS/ZnS QDs used for encapsulation are shown in Figure 1. The morphology of the CdSe@ZnS/ZnS QDs is represented by spherical nanoparticles with an average size around 12 nm, which is characteristic of the giant core–shell QDs structure. The CuInZnS/ZnS sample was dominated by small nanoparticles with an average diameter of around 3 nm. In both systems, the nanomaterial morphology is in good agreement with the reported methods [31,35].

Chiroptically active luminescent CaCO_3_ composites were produced using modified previously reported procedures [36,37], where complex micelles are formed through the combination of a polymer and surfactant, around which CaCO_3_ structures are formed. Briefly, polyvinylpyrrolidone (PVP) and sodium dodecyl sulfate (SDS) are mixed, along with L-cysteine, in a Na_2_CO_3_ solution. To this, aliquots of luminescent (QDs or lanthanides) materials are added. Finally, a solution of CaCl_2_, which contains a further amount of SDS, is added to produce the composites with chiral, luminescent materials embedded within. A range of samples was produced (with the details given in the Section 2 below) and characterized using scanning electron microscopy (SEM), mercury porosimetry, focused ion beam (FIB) microscopy, X-ray diffractometric (XRD) studies, photoluminescence (PL), and diffuse reflectance circular dichroism (DRCD).

### 3.2. Structural Characterisation

**Figure 2:**
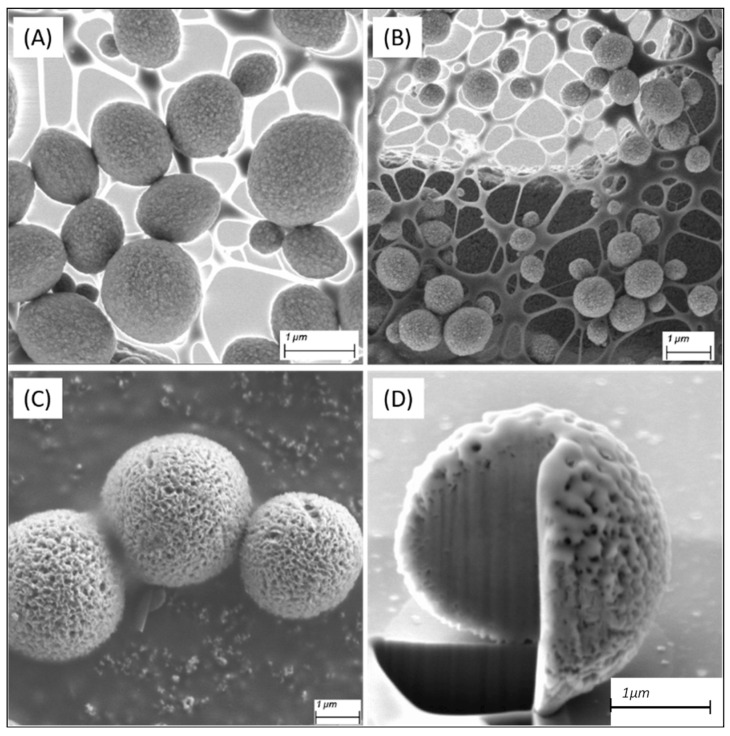
SEM images of the as-synthesized composites loaded with (**A**) CuInZnS/ZnS, (**B**) CdSe@ZnS/ZnS, and (**C**) doped with Eu^3+^, and (**D**) FIB analysis showing a gallium milled cross section of a Eu^3+^-doped CaCO_3_ composite.

Samples were studied via SEM with images given in Figure 2. Each sample demonstrated excellent monodispersity, with mean size distributions given in Supplementary Figure S1. The images show the formation of composites with high monodispersity and average diameters of 1.3 ± 0.2 µm (CuInZnS/ZnS loaded) and 0.9 ± 0.1 µm (CdSe@ZnS/ZnS loaded), and 3.0 ± 0.5 µm for the Eu^3+^-doped composites (Figure S1). Further micrographs of the CaCO_3_ composites are shown in the Supporting Information file (Figures S2–S5). Interestingly, the addition of amino acids was shown to have no noticeable effect upon the diameter or distribution of the composites (SEM images of the achiral composites are shown in Figure S2).

The various loaded composites showed no obvious morphological differences from each other apart from those doped with Eu^3+^, which demonstrated both a marked increase in size and visible porosity (Figure 2C,D). This is to be expected, as while the QDs are simply loaded into the composites, Eu^3+^ has a high partition coefficient for calcite [38] and as such is readily incorporated into its matrix. This charge and size disparity, whereby two Eu^3+^ cations replace three Ca^2+^ cations, creates defects within the microsphere, which is likely the cause of the increased porosity and size. The porosity was analyzed using mercury porosimetry (see Supplementary Figure S6), giving two average size populations of 30 nm and 11 nm for undoped CaCO_3_ while the mean diameter increased to 70 nm upon the addition of Eu^3+^.

Focused ion beam (FIB) analysis (Figure 2D) showed that the composites formed were not hollow, while still being porous throughout. It is expected that some of this visible porosity was lost during the gallium-milling step of FIB, which causes an effect known as `curtaining’ whereby the mill pattern leads to the formation of large periodic strips down the composites [39]. The formation of non-hollow porous composite microspheres is ideal for this work, as they provide a larger surface area within the microsphere to load the luminescent materials.

**Figure 3:**
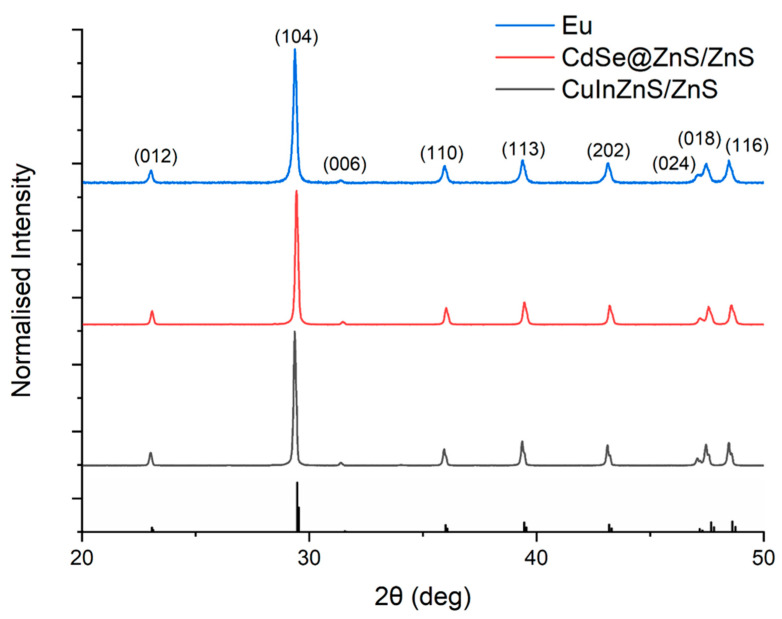
X-ray diffraction pattern of the (blue) Eu^3+^-doped (red), CdSe@ZnS/ZnS, and (black) CuInZnS/ZnS-loaded composites showing the expected peaks for calcite. Reference pattern below: CaCO_3_ calcite phase ICSD 18166.

X-ray powder diffraction (XRD) analysis (Figure 3) showed that all three composite samples demonstrated the characteristic pattern of the CaCO_3_ trigonal calcite phase (space group R-3c n° 167) as reported for similar systems [40].

The main diffractions are related to the (012), (104), (006), (110), (113), (202), (024), (018), and (116) planes of calcite and were observed at 23.31, 29.62, 31.63, 31.14, 39.51, 43.31, 47.24, 47.74, and 48.74 2θ degrees, respectively. The structure refinement (Figure S7) gives a unit cell of 4.9890 and 17.0689 Å for the a and c lattice parameters, respectively. An average crystallite size of 23 nm can be estimated using the Debye–Sherrer equation.

The XRD patterns of CdSe@ZnS/ZnS and CuInZnS/ZnS QDs are shown in Figure S7. The CdSe@ZnS/ZnS QDs XRD pattern (Figure S7A) shows a wurtzite hexagonal structure (space group P63mc n° 186) as observed for similar CdSe QDs [41,42,43]. The diffractions can be assigned to the (100), (002), (101), (102), (2–10), (103), and (2–12) planes of the wurtzite lattice that were observed at 26.3, 27.7, 28.8, 38.6, 46.3, 49.5, 54.9 2θ degrees. The CuInZnS/ZnS QDs XRD pattern is shown in Figure S7B, where the nanoparticles are characterized by a cubic sphalerite (zincblende) structure (space group F-43m n° 216). The main diffraction peaks can be assigned to the (111), (200), (220), and (311) planes that were observed at 48.5, 32.8, 47.6, and 56.0 2θ degrees, respectively. The small peak at 19.5 2θ degrees is often ascribed to a contribution from organic ligands [44]. In both cases, a prominent shift of all the diffractions peaks toward higher angles compared to the CdSe and CuZn_2_InS_4_ phases is consistent with the incorporation of ZnS in the nanoparticles as well-documented for similar hetero-nanostructures produced by shelling or alloying with ZnS [35,45].

Due to the amount of QDs loaded in the CaCO_3_ matrix and the intrinsic broadening of the QDs diffractions due to the nanocrystals’ size, the XRD patterns of the composites produced with CdSe@ZnS/ZnS and CuInZnS/ZnS are completely dominated by the contribution originating from the CaCO_3_ matrix. For this reason, energy dispersive x-ray spectroscopy (EDS) was used to estimate the loading of CaCO_3_ composites. The EDS spectra of CdSe@ZnS/ZnS and CuInZnS/ZnS QDs are shown in Figure S8. The chemical composition analysis of CdSe@ZnS/ZnS QDs shows an atomic ratio of 1:40:14:23 for Cd, Zn, Se, and S respectively. Instead, CuInZnS/ZnS QDs are characterized by an atomic ratio of 1:1.5:20:23 for Cu:In:Zn:S, respectively. Both nanocrystals present a high content of zinc as also expected by their XRD patterns (Figure S8). The EDS spectra of the CaCO_3_ composites (Figure S8) are mainly dominated by the peaks of the matrix, as can be appreciated by the intensity of the Ca Kα peak at 3.69 KeV. The analysis of the Eu^3+^-doped CaCO_3_ shows 3.9% of Eu^3+^, estimated by the intensity of the Eu Lα (5.845 keV) and Eu M (1.131 keV) peaks. The spectra of the QDs-loaded composite show, with meaningful intensity, only peaks related to the main elements present in the nanocrystals such as zinc, sulfur, and selenium. An approximate estimation of the QD loading was performed considering the Zn Lα (1.012 keV) and S Kα (2.037 keV), which resulted in an estimated loading of 4.2% for CdSe@ZnS/ZnS QDs and 5.0% for CuInZnS/ZnS QDs.

### 3.3. Spectroscopic Characterisation

**Figure 4:**
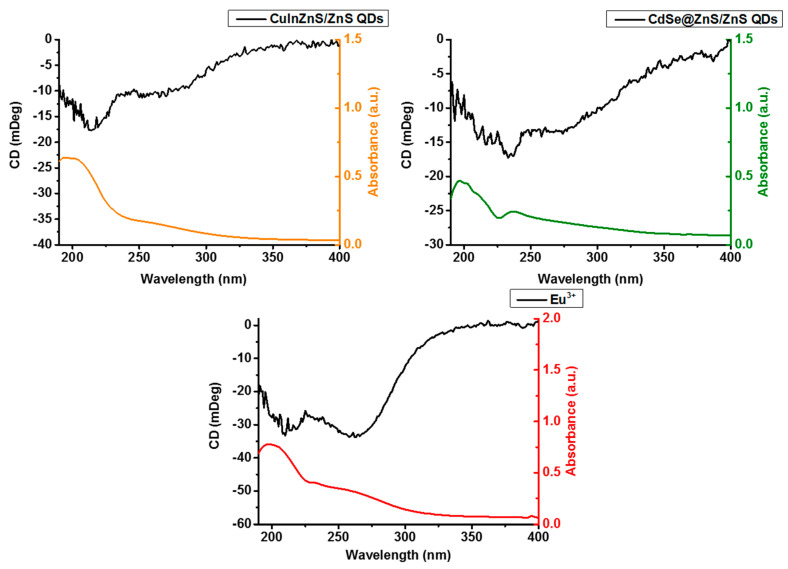
Diffuse reflectance circular dichroism (DRCD) spectroscopy of the CaCO_3_ composites. The black line represents CD spectra while the colored spectra represent the UV-Vis absorbance spectra of the materials.

Diffuse reflectance circular dichroism (DRCD) and UV-Vis spectroscopy were carried out on all materials, as illustrated in Figure 4. A solid-state analytical technique was chosen owing to the poor solubility of CaCO_3_, which limits the effectiveness of analysis in solution. In DRCD, an integrating sphere coated with BaSO_4_ is used to allow the precise solid-state analysis of chiral compounds. BaSO_4_ was the chosen material for coating due to its highly reflective properties; it can reflect light evenly across a wide range of wavelengths. The UV-Vis band positions at 205 and 265 nm correspond to CaCO_3_ [46]. The circular dichroism data of the four samples illustrate the chiral nature of each. There is a dominating CD band that is portrayed by all three spectra in the UV region. This band appears at 220 nm, 230 nm, and 260 nm for the CuInZnS/ZnS QDs, CdSe@ZnS/ZnS QDs, and the Eu^3+^-loaded CaCO_3_ composites, respectively. All the CaCO_3_ composites show a strong absorption in the UV range due to the matrix, and no absorption in the visible range can be appreciated. This is likely due to the modest level of QD loading. L-cysteine is a well-known chiral amino acid, which is often used to impart chirality to nanoparticles [47]. It should be noted that the CD signal differs significantly from the CD spectrum of L-cysteine (See Supplementary Figure S9), proving that the signal is not due to free cysteine. Instead, what is observed is a lack of the Cotton effects, which is typical for chiral molecules (whereby there is a change in optical rotation in the region of maximum absorption). A potential rationalization of this phenomenon is that the amino acid is bonded to CaCO_3_ in such a way that its conformational freedom is highly restricted. In this scenario, the amino acid is incapable of rotating in such a way as to produce Cotton effects, i.e., the ‘switching’ of the chiral signal at the point of maximum absorbance.

FT-IR was also carried out to confirm the presence of cysteine in the composites and is shown in Supplementary Figure S10. Briefly, the main contribution to the FTIR spectra can be related to the calcite matrix. Indeed, the strong broad peak centered around 1450 cm^−1^ is related to the asymmetric C-O stretching ν_3_ band, and two narrow peaks at 870 and 710 cm^−1^ are related to CO_3_ ν_2_ plane deformation and O-C-C bending ν_4_, respectively; the latter is characteristic of crystalline CaCO_3_ [48]. The peaks around 3000 cm^−1^ correspond to the CH_3_ and CH_2_ stretching modes and a broad band related to the COOH functional groups of cysteine [49].

### 3.4. Investigation of Photoluminescence in Eu^3+^-Doped and QD-Loaded CaCO_3_ Composites

**Figure 5:**
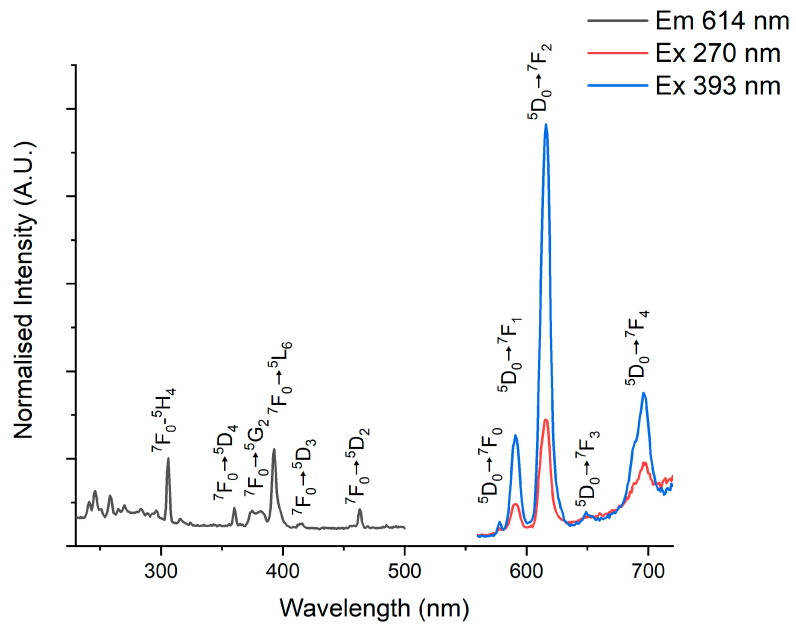
Emission and excitation photoluminescence spectra of Eu^3+^-doped CaCO_3_ composites. Emission was measured using excitation at 270 nm and 393 nm, while excitation was measured using the 614 nm peak, which corresponds to the ^5^D_0_–^7^F_2_ transition.

**Figure 6:**
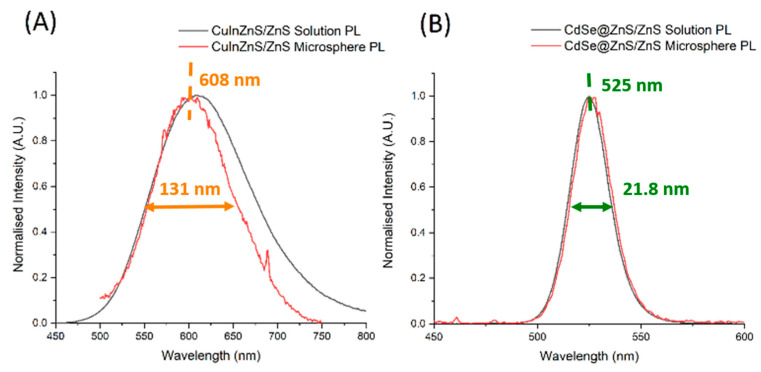
Photoluminescent emission spectra of CuInZnS/ZnS (**A**) and CdSe@ZnS/ZnS (**B**) in solution and loaded into CaCO_3_ composites.

Following the proceeding work, we carried out characterization of the luminescent properties of the Eu^3+^-doped and the CuInZnS/ZnS QD- and CdSe@ZnS/ZnS QD-loaded composites, with the results shown in Figure 5 and Figure 6, respectively.

The excitation photoluminescence spectra (PLEx) and the emission photoluminescence spectra (PLEm) of Eu^3+^-doped CaCO_3_ composites are shown in Figure 5 with the characteristic peaks as has been reported in our previous work and other literature sources [18]. Emission spectra were recorded from 560 nm to 720 nm using an excitation of 393 nm corresponding to ^7^F_0_–^5^L_6_ and using an excitation wavelength of 270 nm corresponding to the absorption energy of the O^2−^/Eu^3+^ charge transfer band (CTB) [50]. Upon excitation at 393 nm, the emission spectrum (Figure 5) is composed of narrow emission lines centered at 579, 590, 614, 650, and 699 nm. They correspond to the ^5^D_0_–^7^F_J_ (J = 0, 1, 2, 3, 4) transitions characteristic of the Eu^3+^ ions. The strongest peak is located at 614 nm, which can be related to the ^5^D_0_–^7^F_2_ transition [50]. As common for similar systems [50], the two strongest peaks at 589 and 614 nm correspond to the ^5^D_0_–^7^F_1_ and ^5^D_0_–^7^F_2_ transitions, respectively. While the former is magnetic dipole-allowed and local symmetry-independent, the latter is an induced dipole transition and is strongly dependent on local symmetry.

Photoluminescent spectra of the QD-loaded samples compared to the original QD PL in toluene solution are shown in Figure 6. The CuInZnS/ZnS QD-loaded CaCO_3_ demonstrated a broad emission profile with a full width at half maximum (FWHM) of 131 nm, peaking at 608 nm, which is indicative of this type of QD. Indeed, as reported by Castro et al., copper indium sulfide quantum dots are characterized by a large Stokes shift and FWHM in the order of hundreds of nanometers [51]. Due to their detailed analysis of the origin of these optical features, the authors established that multiple intraband defects are involved in the radiative recombination via a donor–acceptor recombination mechanism. CdSe@ZnS/ZnS QD-loaded CaCO_3_ showed a much sharper emission peak with an FWHM of 21.8 nm centered on 525 nm, which is well-documented for the emission of II-VI QDs, which is dominated by a band-to-band recombination mechanism [31].

When comparing the QD-loaded composites’ PL to the PL of the original solutions, no significant red-shifting of the peak position is noticeable upon incorporation inside the CaCO_3_ lattice for either sample, with the CuInZnS/ZnS in fact showing a minor blue shift. The absence of red-shifting indicates that both QDs were incorporated singly throughout the composites’ structures and have avoided aggregation, which leads to strong red-shifting in the PL emission peak.

To further characterize the effects of composite loading upon the PL emission, the fluorescence lifetime was measured for each sample and compared to the original QD prior to incorporation (Table 1). CdSe@ZnS/ZnS QD in toluene solution showed a monoexponential decay with a lifetime of 14.4 ns; this value is in agreement with the observation reports on CdSe QDs shelled with ZnS [31,52]. The photoluminescent decay of CuInZnS/ZnS QDs were fit with a triexponential decay to take into account multiple types of defects that can contribute to the emission via a donor–acceptor recombination mechanism [51]. An average lifetime of 434 ns is estimated; average lifetime values in the order of few hundreds of nanoseconds are in line with the behavior of copper indium sulfide and zinc alloyed systems [53,54]. In both cases, the QDs displayed a minor decrease in PL lifetime after the incorporation in CaCO_3_. CdSe@ZnS/ZnS QDs showed a decrease from an average time of 14 ns in solution to 6.4 ns in the CaCO_3_, while CuInZnS/ZnS QDs showed a more significant decrease from 434 to 295 ns. Therefore, this indicates increased de-excitation, which can be explained via binding with cysteine molecules, which passivate the QD surface less effectively than the original ligands. This may also be related to defects at the QDs–CaCO_3_ interface, likely due to lattice mismatching that can act as a new pathway for non-radiative recombination process, causing the observed reduction in lifetimes.

For Eu^3+^-doped calcium carbonate composites, the measured lifetime values were fitted using biexponential decay and were found to be T_1_, = 0.398 ms and T_2_, = 1.488 ms, with T_ave_ = 0.635 ms using the excitation wavelength of 393 nm. The results are well in line with previous reports for Eu^3+^-doped calcium carbonate composites [18].

## 4. Conclusions

Thus, we have successfully prepared several types of new multimodal CaCO_3_ nanocomposites. Initially, achiral CaCO_3_ composites were synthesized using a modified reported procedure. Their subsequent use of amino acids imparted a unique chiroptical activity and CD signal to these structures for the first time. The CD activity itself presents a significant research interest, as its extension to the upper wavelengths seems to point to a type of ‘chiral scattering’ or ‘chiral complexation’. Using this observation, the occurrence of chiral encapsulation can be determined using CD spectroscopy.

These composites have subsequently been used to encapsulate various luminescent materials. The encapsulation of CdSe@ZnS/ZnS and CuInZnS/ZnS QDs imparted green and orange fluorescence, respectively, whilst Eu^3+^ was used to produce red photoluminescent doped CaCO_3_, with these emission spectra studied in detail.

With the addition of this functionality, we have demonstrated the intrinsic multimodal nature of this new class of chiral composites via the loading of specific ions or nanoparticles. Overall, this work is an important example of the new approaches to multimodal chiroptically active materials, which are expected to attract much attention and find a range of applications in the coming years.

## Figures and Tables

**Table 1:** Fluorescence lifetimes of QDs and QD-loaded composites.

Sample	T_1_ (ns)	A_1_ (a.u)	T_2_ (ns)	A_2_ (a.u)	T_3_ (ns)	A_3_ (a.u)	T_ave_ (ns)
CdSe@ZnS/ZnS QDs solution	14.4	-	-	-	-	-	-
CdSe@ZnS/ZnS QDs in CaCO_3_	6.4	-	-	-	-		-
CuInZnS/ZnS QDs solution	163	0.2287	335	0.5854	1080	0.1859	434
CuInZnS/ZnS QDs in CaCO_3_	71.5	0.142	335	0.8502	8.32	0.0074	295

## Data Availability

Data are available in section “MDPI Research Data Policies” at https://www.mdpi.com/ethics (accessed on 26 December 2023).

## Supplementary Materials

The following supporting information can be downloaded at: https://www.mdpi.com/article/10.3390/nano14010100/s1, Figure S1. Size distributions of (A) CuInZnS/ZnS (B) CdSe@ZnS/ZnS loaded and (C) Eu^3+^ doped microspheres where *N* = 160, 150 and 175, respectively. Figure S2. Achiral CaCO_3_ composites. Figure S3. SEM images of CaCO_3_ composites loaded with CuInZnS:ZnS QDs. Figure S4. SEM images of CaCO_3_ composites loaded with CdSe@ZnS/ZnS QDs. Figure S5. SEM images of Eu^3+^ doped CaCO_3_ composites. Figure S6. Mercury porosimetry measurements of doped and undoped CaCO_3_ composites. Figure S7. (A) XRD pattern of CdSe@ZnS/ZnS QDs, reference pattern: CdSe wurtzite phase (black) ICSD 415784 and ZnS wurtzite phase (red) 67453. (B) XRD pattern of CuInZnS/ZnS QDs, reference patterns: CuZn_2_InS_4_ sphalerite phase (black) and ZnS sphalerite phase (red). The asterisk marks the residual contribution from organic ligands. Figure S8. EDS spectra of CdSe@ZnS/ZnS, CuInZnS/ZnS QDs and the different CaCO_3_ composites. Figure S9. CD spectrum of *L*-cysteine in water. Figure S10. FT-IR spectra of the composites loaded with (A) CuInZnS/ZnS, (B) CdSe@ZnSZnS and (C) doped with Eu^3+^ illustrating the presence of cysteine as evidenced by its characteristic peaks at 3000 cm^−1^.
