# Supplementary material for: Chiroptically Active Multi-Modal Calcium Carbonate-Based Nanocomposites
Source: Nanomaterials (Basel). 2023 Dec 31;14(1):100. doi: 10.3390/nano14010100 (PMC10780737; doi:10.3390/nano14010100)
Supplement: Supplementary file 1 [file nanomaterials-14-00100-s001.zip › nanomaterials-2750355-supplementary.pdf]

Supporting Information

Chiroptically Active Multi-Modal Calcium Carbonate-Based Nanocomposites

Fearghal C. Donnelly,[1,2] Finn Purcell-Milton,[3] Eoin Caffrey,[4] Lorenzo Branzi, [1] Shelley Stafford,[1] Faisal Ali Alhammad,[1] Olan Cleary,[1] Munirah Ghariani, [1] Vera Kuznetsova,[1] and Yurii K. Gun'ko[*1,2]

[1] School of Chemistry, Trinity College Dublin, D02PN40, Dublin, Ireland.
[2] BiOrbic Bioeconomy SFI Research Centre , University College Dublin, D04 F438, Dublin, Ireland
[3] Chemical & BioPharmaceutical Science, Technological University Dublin, Grangegorman, D07 H6K8, Dublin, Ireland
[4] School of Physics, Trinity College Dublin, D02PN40, Dublin.

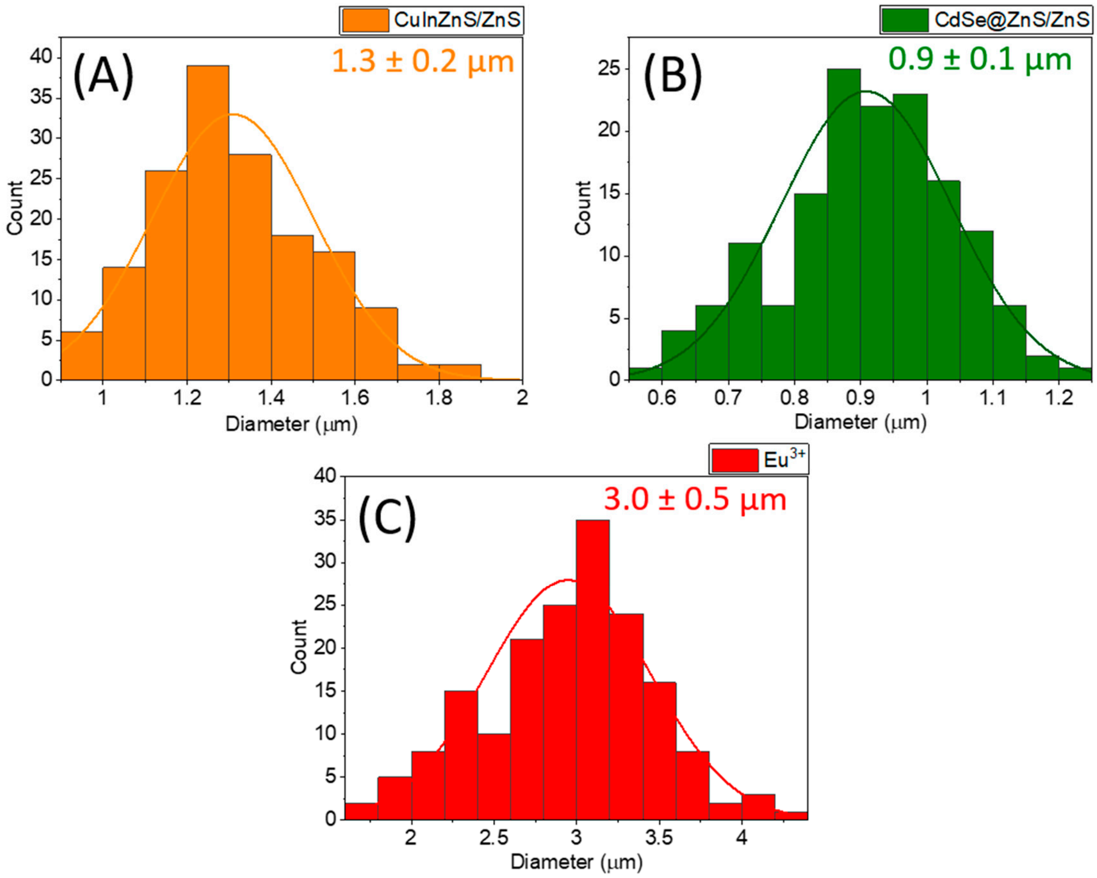

Figure S1. Size distributions of (A) CuInZnS/ZnS (B) CdSe@ZnS/ZnS loaded and (C) Eu^{3+} doped microspheres where *N* = 160, 150 and 175, respectively. .

Additional SEM Images

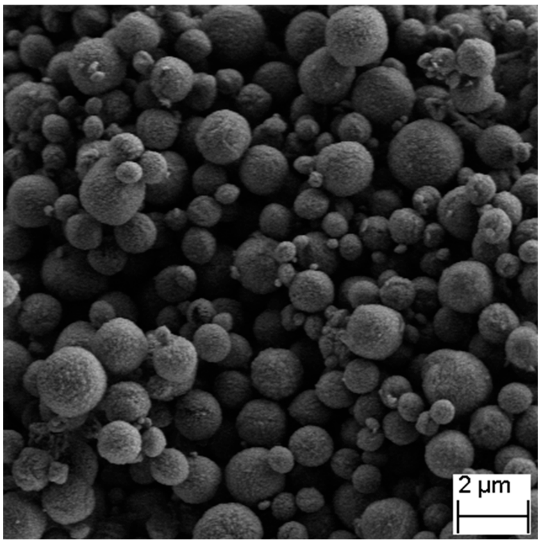

Figure S2. Achiral $CaCO_3$ composites.

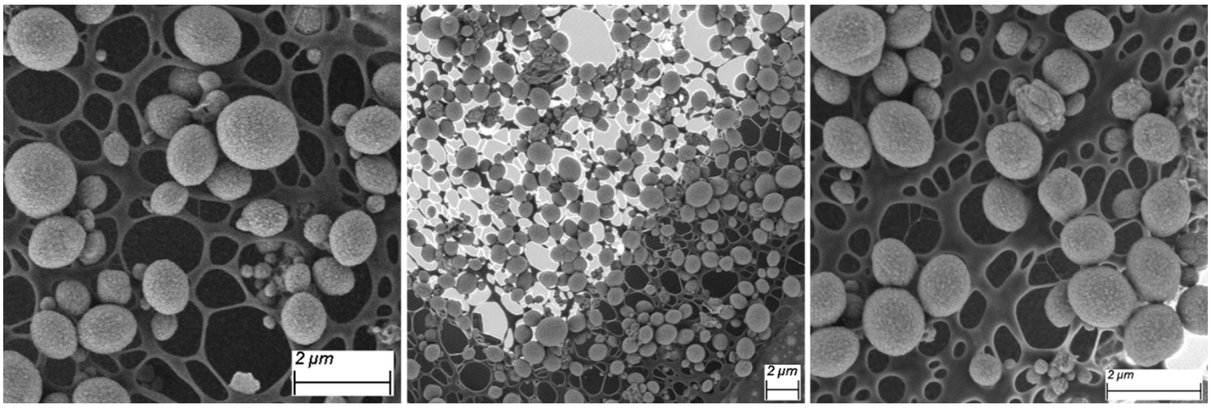

Figure S3. SEM images of $CaCO_3$ composites loaded with CuInZnS:ZnS QDs.

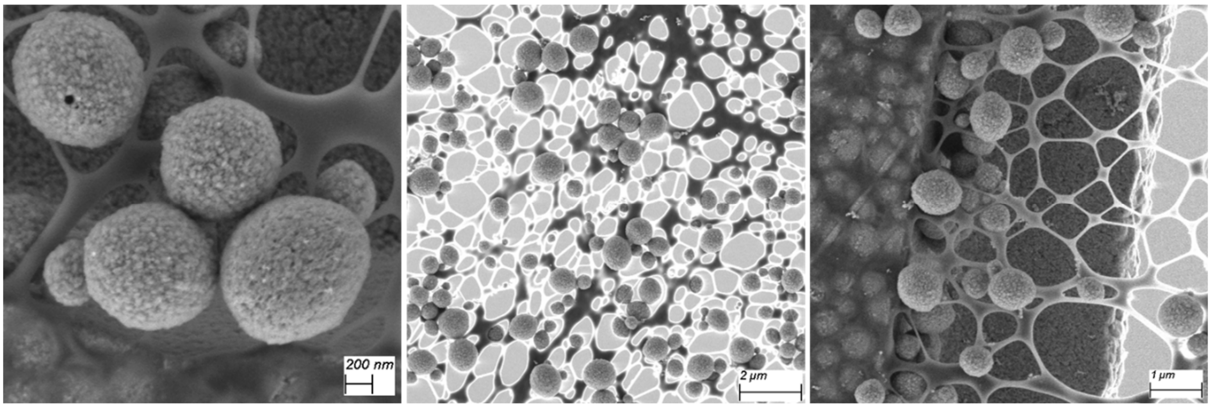

Figure S4. SEM images of $CaCO_3$ composites loaded with CdSe@ZnS/ZnS QDs.

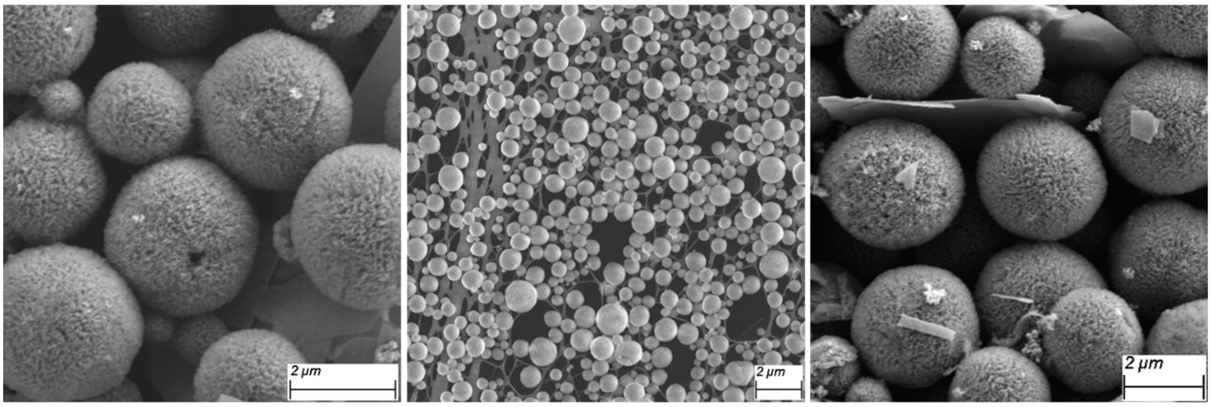

Figure S5. SEM images of Eu^{3+} doped $CaCO_3$ composites.

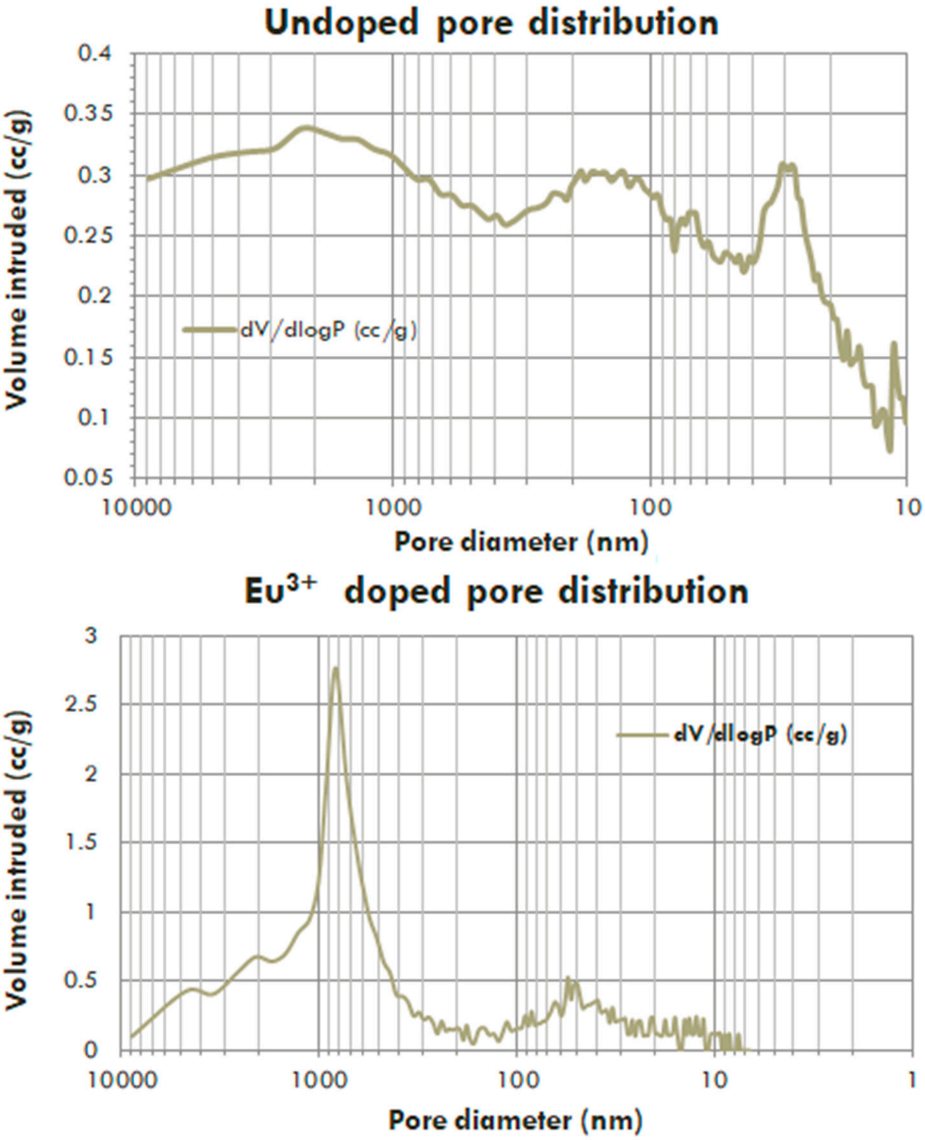

Figure S6. Mercury porosimetry measurements of doped and undoped $CaCO_3$ composites. In the undoped composites there are two main pore sizes of 30 and 11 nm. In the doped samples, the

average pore diameter is c. 70 nm. In both plots, the large peaks at higher diameters correspond to voids between the microspheres and can therefore be discounted.

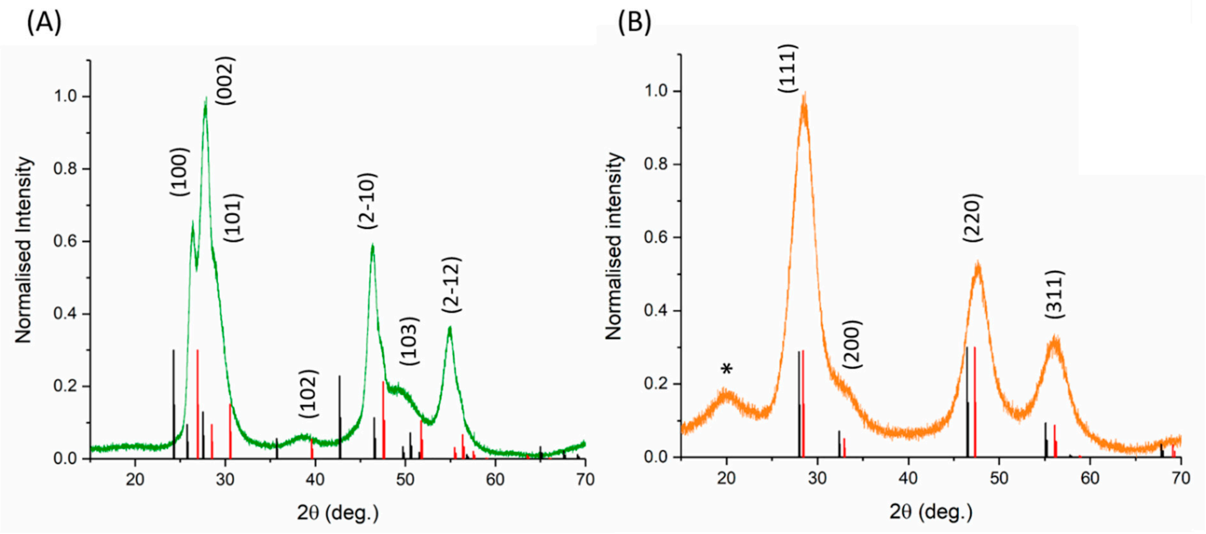

Figure S7. A) XRD pattern of CdSe@ZnS/ZnS QDs, reference pattern: CdSe wurtzite phase (black) ICSD 415784 and ZnS wurtzite phase (red) 67453. B) XRD pattern of CuInZnS/ZnS QDs, reference patterns: $CuZn_2InS_4$ sphalerite phase (black) and ZnS sphalerite phase (red). The asterisk marks the residual contribution from organic ligands.

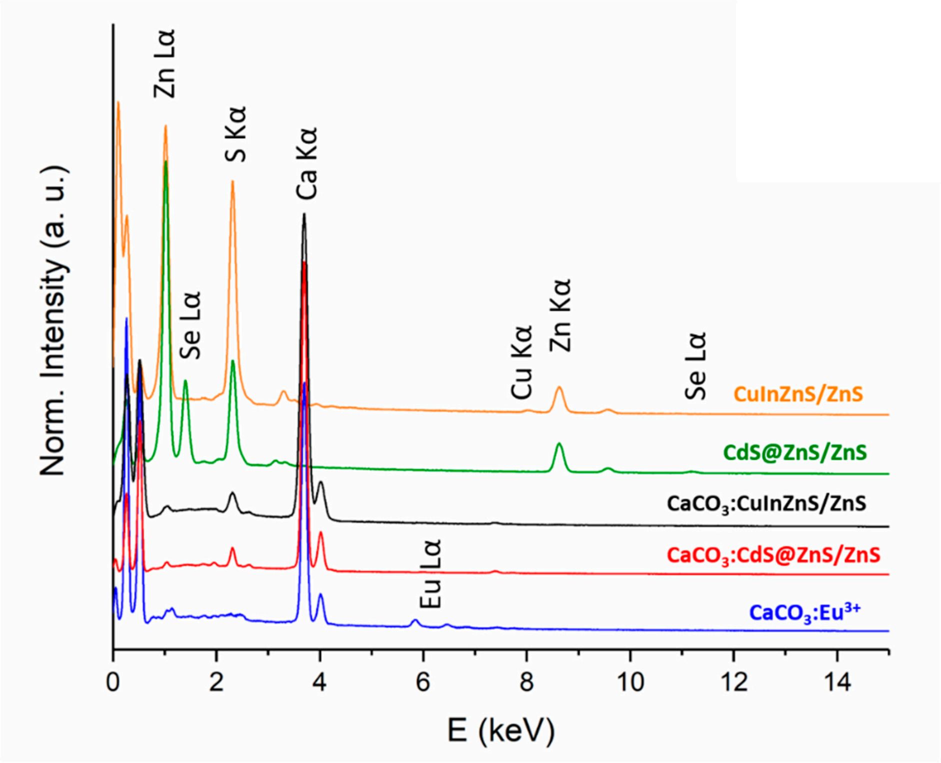

Figure S8. EDS spectra of CdSe@ZnS/ZnS, CuInZnS/ZnS QDs and the different CaCO3 composites.

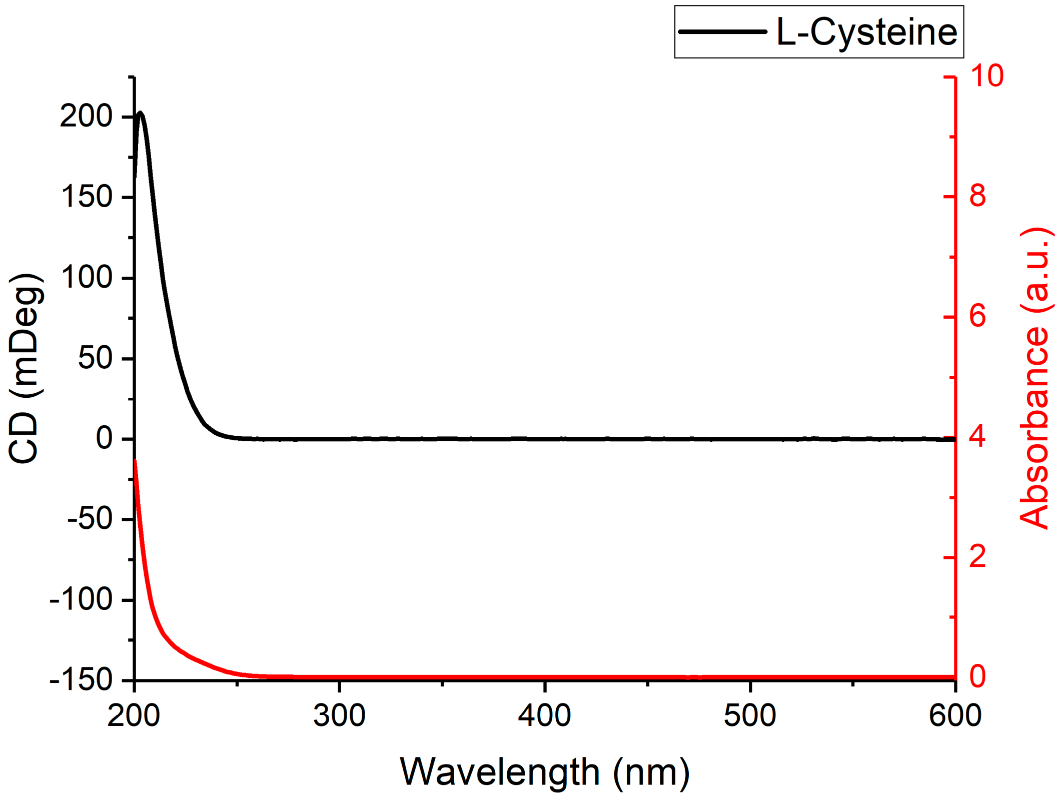

Figure S9. CD spectrum of *L*-cysteine in water.

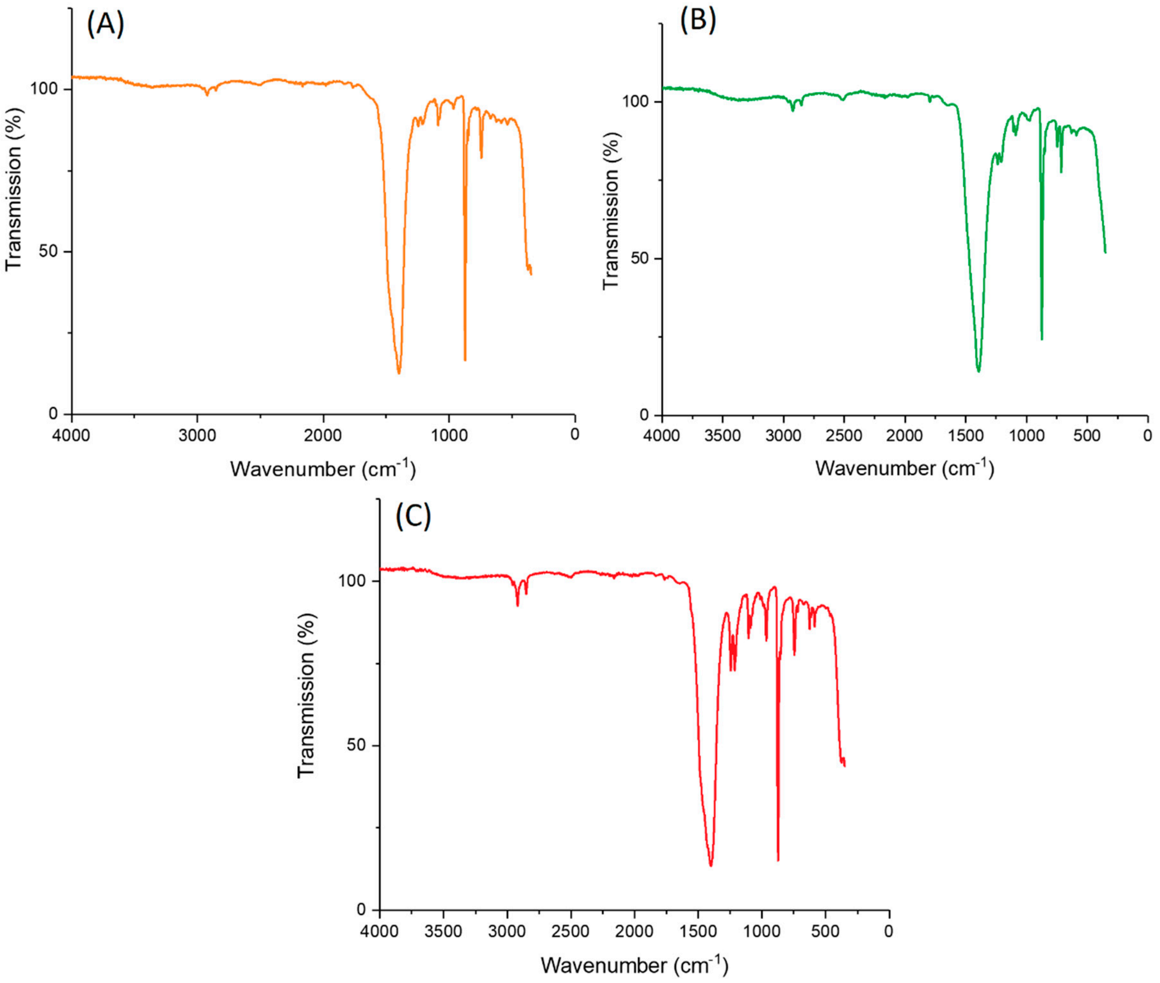

Figure S10. FT-IR spectra of the composites loaded with (A) CuInZnS/ZnS, (B) CdSe@ZnSZnS and (C) doped with Eu^{3+} illustrating the presence of cysteine as evidenced by its characteristic peaks at 3000 cm^{-1}.
